# An Uncommon Presentation of Crowned Dens Syndrome Without Systemic Inflammation

**DOI:** 10.7759/cureus.84853

**Published:** 2025-05-26

**Authors:** Pavan Sakhamuru, Manav Nayeni, Ryan Nazari, Kazi Syed, Kenneth Miller

**Affiliations:** 1 College of Osteopathic Medicine, Kansas City University, Kansas City, USA; 2 Physical Medicine and Rehabilitation, Kansas City Veterans Affairs (VA) Medical Center, Kansas City, USA

**Keywords:** atlanto-axial joint, calcium pyrophosphate dihydrate crystal deposition, cds, cervical spine anomalies, chronic neck pain, crowned dens syndrome, odontoid process

## Abstract

Crowned Dens Syndrome (CDS) is a rare but important consideration in the differential diagnosis of cervical spine pain in older adults. CDS is characterized by calcium pyrophosphate dihydrate (CPPD) crystal deposition around the odontoid process, often leading to symptoms that overlap with more common conditions such as rheumatoid arthritis (RA), meningitis, or cervical spondylosis. We report the case of a 74-year-old male with chronic neck pain and restricted cervical range of motion. Advanced imaging revealed characteristic calcifications surrounding the odontoid process, pannus formation, and erosive changes at the C1-C2 articulation, consistent with CDS. Clinical evaluation supported a diagnosis of CPPD-related CDS. The patient was successfully managed conservatively with nonsteroidal anti-inflammatory drugs and physical therapy, with notable symptomatic improvement over time. This case reinforces the utility of CT imaging in diagnosing CDS and differentiating it from other inflammatory or degenerative cervical conditions. Awareness of CDS and a high index of suspicion are essential for early diagnosis, appropriate treatment, and avoidance of unnecessary interventions. Conservative management remains effective in most cases; however, further studies are necessary to evaluate alternative therapies for refractory presentations.

## Introduction

Crowned Dens Syndrome (CDS) is a rare and often underdiagnosed condition that contributes to acute cervical pain commonly seen in the elderly population [1]. It is characterized by calcium pyrophosphate dihydrate (CPPD) crystal deposits encircling the odontoid process (dens) of the C2 vertebra [1]. Clinical manifestations primarily include acute cervical pain, restricted range of motion (ROM), and a local inflammatory response, which may cause fever and neurological symptoms, raising concern for an infectious or autoimmune process [2]. Moreover, in patients with chronic, indolent cervical symptoms, CDS is unlikely to be included in the differential diagnosis. Instead, more common conditions such as cervical spondylosis, osteoarthritis, or muscle strain are typically considered. Given the diagnostic challenges, overlapping clinical presentations, and potential consequences of misdiagnosis, determining the precise etiology of axial neck pain in elderly patients is essential. An accurate diagnosis allows for timely, targeted treatment and helps avoid unnecessary surgical intervention or mismanagement of potentially serious alternative conditions. This case report highlights the importance of integrating imaging, clinical evaluation, and multidisciplinary consultation to optimize outcomes in patients with suspected CDS.

## Case presentation

A 74-year-old male presented to the Physical Medicine and Rehabilitation clinic with complaints of chronic neck pain and stiffness for approximately one year. His past medical history included type 1 diabetes mellitus, hypothyroidism, hypertension, hyperlipidemia, stage 2 chronic kidney disease, obstructive sleep apnea, carpal tunnel syndrome, and cervical myelopathy status post C3-4 discectomy/fusion approximately six years prior. The patient described his symptoms as a shooting pain originating at the left base of the neck and radiating down the left shoulder. This pain was positionally dependent and significantly worsened with neck rotation to the left. He denied numbness or tingling in either arm. He did endorse some balance difficulties and used a cane for ambulation support. The patient reported dramatic improvement in both pain and range of motion with the use of naproxen 500 mg twice daily as needed.

The neuromuscular examination was largely unremarkable, apart from slightly brisk brachioradialis and biceps reflexes bilaterally. Cervical spine examination revealed forward neck posture, restricted lateral flexion, severely limited cervical rotation, and tenderness over the left suboccipital muscles. Cervical extension, bilateral rotation, and bilateral side-bending demonstrated 4/5 strength, while cervical flexion and all other upper extremity strength testing were 5/5 bilaterally. Hoffman’s, Clonus, and Spurling’s tests were negative bilaterally.

**Figure 1:**
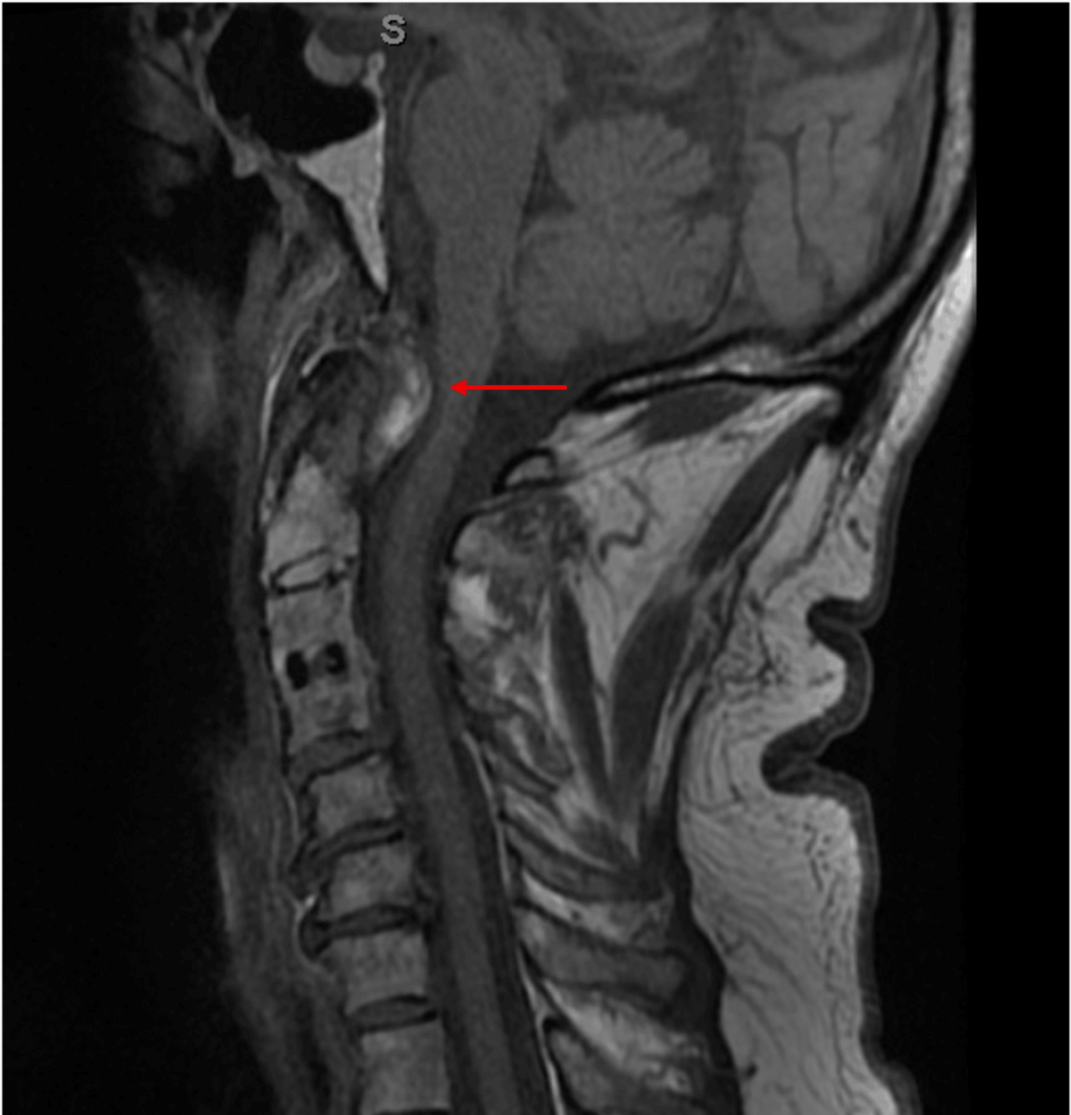
T1-weighted sagittal MRI of the cervical spine. The red arrow indicates pannus formation at the level of the dens.

Given the duration of the patient’s symptoms and cervical spine physical exam findings, cervical spinal imaging was ordered. MRI of the cervical spine revealed pannus formation at the level of the dens, mild C4-5 spinal stenosis, multilevel facet and uncovertebral degenerative changes, and C4 myelomalacia (Figure 1).

**Figure 2:**
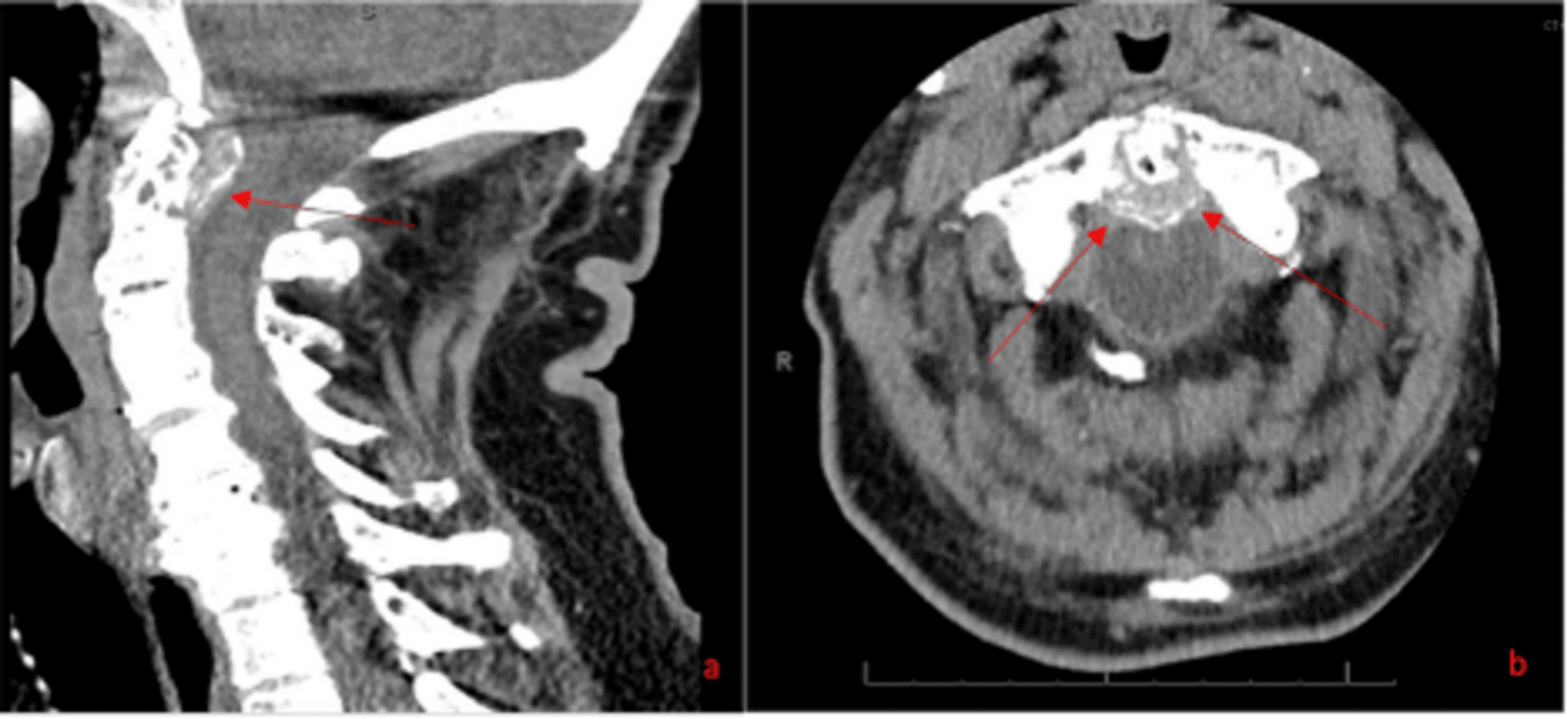
Sagittal (a) and axial at the level of C1 (b) views of the cervical spine on CT scan without contrast. Red arrows point to the crowned dens in the different views. Also visualized are the extensive erosive changes involving the odontoid process and base of the dens.

Due to MRI’s limited sensitivity in detecting calcific deposits, particularly around the odontoid process, the findings were inconclusive. With concern for CDS, a non-contrast cervical CT was subsequently recommended and obtained. The CT demonstrated marked thickening of the retro-odontoid soft tissue with erosive changes at the C1-C2 articulation, along with scattered calcifications (Figure 2).

Further evaluation by rheumatology determined that the clinical picture was consistent with CPPD arthritis secondary to osteoarthritis. Though ESR and CRP were considered, inflammatory markers were not obtained, as systemic inflammation was deemed unlikely due to the absence of synovitis or inflammation in the peripheral joints. The rheumatologist recommended continued conservative management, given the patient’s prior positive response to naproxen. A neurosurgical consultation was also initiated to evaluate the need for surgical intervention. The neurosurgeon concurred with the conservative approach, suggesting continued use of naproxen, physical therapy, and possible steroid injections.

Based on the imaging findings and consult recommendations, the patient continued with as-needed naproxen therapy, which had previously provided symptom relief. Physical therapy, including cervical stretching and strengthening, transcutaneous electrical nerve stimulation (TENS), soft tissue therapy, and dry needling, contributed to gradual improvement in cervical range of motion and pain control. The patient remains clinically stable with ongoing conservative management.

## Discussion

This case presents a unique example of how CPPD-related CDS, although rare, should be included in the differential diagnosis for chronic indolent neck pain in an elderly patient without classic systemic or inflammatory symptoms, especially in the presence of previous cervical spine pathologies [2-4]. CDS remains a rare cause of acute neck pain complaints [2]. However, a 2023 retrospective analysis conducted across eight academic hospitals in Japan demonstrated an average of 4.6 cases of CDS per hospital over a two-year period, indicating that CDS may be more prevalent than previously expected [5]. Typically, CPPD is considered a peripheral joint disease [6-7]; however, almost 24% of CPPD cases can involve the spinal column [8]. The theorized mechanism by which CPPD contributes to CDS is crystal deposition in the articular cartilage and periarticular tissues, particularly in the transverse ligaments, alar ligaments, and structures adjacent to the odontoid process [9-10]. The calcifications around the odontoid process contribute to mechanical irritation and disruption of surrounding neurovascular structures, leading to pain and inflammation [11].

Demographically, CDS most commonly affects women over 65 years of age and frequently presents as acute or recurrent episodes of neck pain and stiffness. It is often associated with fever, leukocytosis, and elevated inflammatory markers, with approximately 88.3% of patients with CDS exhibiting such elevations [1,7]. Delirium is also well-documented in elderly patients during acute presentations [1]. Thus, many cases of potential CDS are likely overlooked or misattributed to more common pathologies including meningitis, giant cell arteritis, rheumatoid arthritis (RA), polymyalgia rheumatica, cervical spondylitis, tumors, fever of unknown origin, or even stroke, especially when neurologic symptoms are prominent [12]. Cervical spondylosis was considered unlikely in this patient due to minimal to no reduction in neck muscle strength. Interestingly, in this case, the patient was afebrile, did not have leukocytosis, and did not demonstrate other systemic inflammatory symptoms. Therefore, the presentation can be interpreted as a unique case of chronic indolent CDS without significant systemic or inflammatory features.

Literature has shown that CT offers superior detection of calcifications and is the preferred method for diagnosing CDS [13]. Diagnostic imaging for CDS can be further enhanced with dual-energy CT, which identifies calcium deposits with greater sensitivity [14]. Interestingly, atlantoaxial calcifications have been observed in asymptomatic individuals, particularly the elderly. Studies have reported calcifications in up to 34% of patients over 60 years old and 49% of those over 80 years old undergoing CT imaging for unrelated reasons [15]. This further complicates the clinical picture; therefore, imaging findings must always be interpreted in the context of patient symptoms. 

In our patient, symptoms were dramatically improved with naproxen. Evidence has shown the effectiveness of NSAIDs in managing symptoms related to CDS, with physical therapy serving as a helpful adjunct for CPPD-related diseases [7,11,16]. Additionally, some literature demonstrates the efficacy of oral glucocorticoids, such as low-dose prednisone, in reducing acute inflammation and pain [17-18]. Axial steroid injections can also be considered as a treatment option. While no high-quality evidence from large-scale studies exists, a 2009 case report demonstrated some efficacy of corticosteroid injections in managing refractory CDS-related neck pain [19]. In more refractory cases, cytokine-targeted agents such as anakinra have been used [20].

## Conclusions

Although rare from an epidemiological standpoint, CDS is an important cause of acute or chronic neck pain and restricted range of motion, particularly in elderly patients. Early detection and treatment of CDS can significantly improve patient outcomes. Patients should undergo cervical CT, the most appropriate imaging modality, to evaluate for CDS. Appropriate laboratory testing should also be performed to rule out other inflammatory or infectious processes if fever or polyarthritis is present. For patients with concomitant inflammatory conditions, a multidisciplinary approach, including input from rheumatology, is recommended for optimal management. Whenever possible, conservative treatment with NSAIDs and low-dose steroids should be combined with physical therapy. Additional research should explore the efficacy of axial steroid injections as a potential targeted treatment option. Ultimately, timely identification and appropriate intervention led to a positive outcome for our patient. CDS has a good prognosis and can be effectively treated when diagnosed and managed appropriately.
